# Supplementary material for: Whole-Exome Sequencing in Family Trios Reveals De Novo Mutations Associated with Type 1 Diabetes Mellitus
Source: Biology (Basel). 2023 Mar 7;12(3):413. doi: 10.3390/biology12030413 (PMC10044903; doi:10.3390/biology12030413)
Supplement: Supplementary file 1 [file biology-12-00413-s001.zip › biology-2155435-supplementary.pdf]

Whole exome sequencing in family trios reveals *de novo* mutations associated to Type 1 Diabetes Mellitus

Supplementary Material

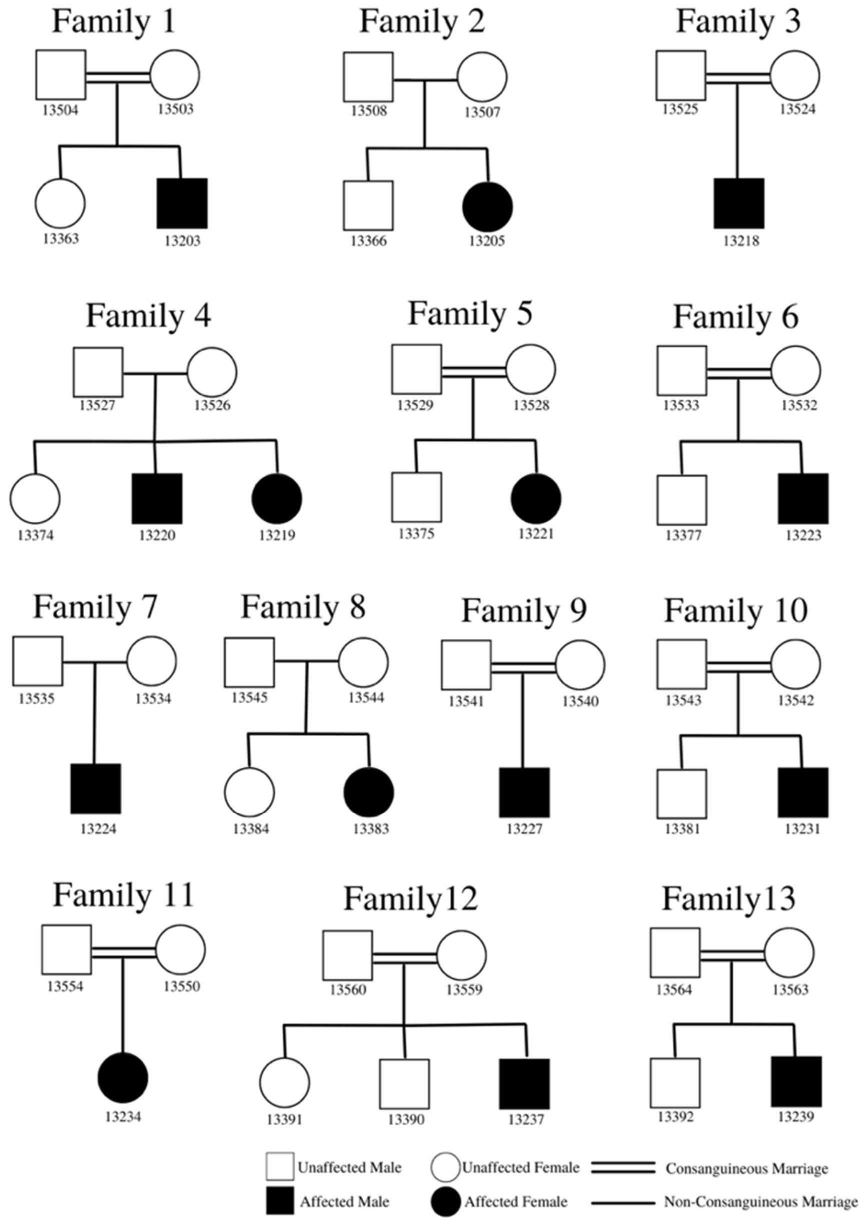

Figure S1. Pedigree of the 13 Emirati case-parent trios.

Table S1. Sequencing coverage, number of reads and additional metrices for exomes for the proband, mother, father and sibling individuals.

Family #	Sample	Mean coverage	# of Reads	# of Mapped reads	% of Mapped reads	>=10X	>=20X
Family 1	13203P	44.4706X	44,573,219	44,538,258	99.92%	92.13%	78.37%
	13503M	41.8621X	42,973,763	42,927,322	99.89%	91.52%	76.27%
	13504F	40.6472X	42,019,107	41,924,175	99.77%	90.62%	74.22%
	13363S	44.7392X	45,790,176	45,744,269	99.90%	91.99%	78.04%
Family 2	13205P	34.0393X	44,029,273	43,712,841	99.28%	90.13%	70.78%
	13507M	44.4842X	60,886,912	60,070,914	98.66%	95.05%	83.79%
	13508F	39.4141X	54,475,705	53,865,334	98.88%	92.80%	78.79%
	13366S	35.9664X	48,654,472	45,325,791	93.16%	91.74%	74.34%
Family 3	13218P	27.9233X	31,524,936	31,413,713	99.65%	81.88%	56.88%
	13524M	35.0133X	36,776,801	36,693,713	99.77%	86.84%	67.51%
	13525F	29.7514X	31,549,529	31,497,836	99.84%	84.29%	60.87%
Family 4	13219P	34.5633X	45,493,043	45,044,053	99.01%	92.27%	74.27%
	13220P	30.8297X	44,550,505	44,434,643	99.74%	91.51%	71.43%
	13526M	34.1842X	45,783,196	44,771,053	97.79%	90.63%	71.25%
	13527F	32.3396X	50,968,036	49,901,710	97.91%	89.48%	69.10%
	13374S	32.3485X	51,307,523	50,062,947	97.57%	89.90%	70.14%
Family 5	13221P	50.8449X	47,361,478	47,324,289	99.92%	94.07%	85.89%
	13528M	31.5975X	32,379,009	32,271,549	99.67%	85.38%	63.52%
	13529F	5.1495X	8,361,772	8,307,931	99.36%	11.98%	3.51%
	13375S	35.1454X	36,586,708	36,401,344	99.49%	88.36%	69.12%
Family 6	13223P	31.8506X	32,571,692	32,448,883	99.62%	90.09%	70.67%
	13532M	52.7466X	53,664,622	53,510,877	99.71%	93.11%	82.27%
	13533F	40.3865X	42,823,876	42,757,439	99.84%	90.32%	73.87%
	13377S	35.3281X	39,920,228	39,232,613	98.28%	92.08%	73.64%
Family 7	13224P	40.3797X	43,649,840	43,570,675	99.82%	90.88%	74.47%
	13534M	42.911X	43,592,007	43,533,804	99.87%	92.13%	78.40%
	13535F	46.7866X	47,112,724	47,075,564	99.92%	92.77%	80.26%
Family 8	13383P	33.2355X	55,152,965	53,376,503	96.78%	90.81%	71.67%
	13544M	20.2205X	33,328,313	32,985,589	98.97%	78.13%	44.14%
	13545F	22.0122X	36,515,316	34,861,097	95.47%	82.26%	49.37%
	13384S	22.0624X	34,365,054	33,721,504	98.13%	78.64%	47.86%
Family 9	13227P	27.9016X	49,368,189	45,404,341	91.97%	88.88%	63.23%
	13540M	35.9824X	51,826,041	51,247,451	98.88%	93.31%	78.36%
	13541F	20.6901X	28,862,825	28,166,170	97.59%	81.20%	46.14%
Family 10	13231P	47.0469X	48,117,534	47,996,081	99.75%	93.14%	81.14%
	13542M	44.8075X	46,162,815	46,134,715	99.94%	91.77%	78.24%
	13543F	38.7933X	40,104,482	40,065,095	99.90%	89.95%	73.09%
	13381S	87.7898X	96,866,420	96,258,809	99.37%	97.48%	93.97%
Family 11	13234P	36.4809X	53,146,982	52,761,974	99.28%	93.29%	78.87%
	13550M	45.3447X	50,905,533	50,773,024	99.74%	93.25%	80.22%
	13554F	45.8167X	51,178,715	51,101,598	99.85%	92.83%	79.35%
Family 12	13237P	106.6393X	115,769,038	115,600,099	99.85%	97.98%	95.54%
	13559M	107.6248X	115,857,332	115,738,486	99.90%	97.69%	95.16%
	13560F	64.4619X	59,960,773	59,911,629	99.92%	95.17%	88.76%
	13390S	107.6248X	115,857,332	115,738,486	99.90%	97.69%	95.16%
	13391S	77.4018X	80,116,885	80,071,062	99.94%	96.68%	92.05%
	13239P	73.6161X	80,476,962	80,388,393	99.89%	97.03%	92.58%

Family 13	13563M	37.4867X	52,893,298	52,356,998	98.99%	92.19%	76.28%
	13564F	98.9313X	102,927,970	102,519,904	99.60%	97.60%	94.39%
	13392S	71.0402X	76,233,436	76,158,776	99.90%	97.01%	92.44%
		Average = 45.3743X			Average = 99.00%	Average = 89.56%	Average =73.99%

P: Proband; M: Mother; F: Father; S: Sibling

Table S2. All detected SNPs and indels before applying filtering pipeline in each.

Family Number	Sample	Total SNP Count	Total INDEL Count	Unknown
Family 1	13203P	3108	1395	0
Family 2	13205P	2614	1376	77
Family 3	13218P	4318	1661	102
Family 4	13219P + 13220P	668	201	6
Family 5	13221P	3245	1041	73
Family 6	13223P	3033	1192	88
Family 7	13224P	5229	2270	150
Family 8	13383P	15142	2605	92
Family 9	13227P	6552	1985	105
Family 10	13231P	3326	1589	112
Family 11	13234P	5414	2284	123
Family 12	13237P	17870	3375	152
Family 13	13239P	4388	2490	106
	Total	74907	23464	1186

SNP: single nucleotide polymorphism; INDEL: insertion/deletion; Unknowns: neither.
